# Genome-wide copy number variations in *Oryza sativa* L.

**DOI:** 10.1186/1471-2164-14-649

**Published:** 2013-09-23

**Authors:** Ping Yu, Cai-Hong Wang, Qun Xu, Yue Feng, Xiao-Ping Yuan, Han-Yong Yu, Yi-Ping Wang, Sheng-Xiang Tang, Xing-Hua Wei

**Affiliations:** 1 State Key Laboratory of Rice Biology, China National Rice Research Institute, Hangzhou, China

## Abstract

**Background:**

Copy number variation (CNV) can lead to intra-specific genome variations. It is not only part of normal genetic variation, but also is the source of phenotypic differences. Rice (*Oryza sativa* L.) is a model organism with a well-annotated genome, but investigation of CNVs in rice lags behind its mammalian counterparts.

**Results:**

We comprehensively assayed CNVs using high-density array comparative genomic hybridization in a panel of 20 Asian cultivated rice comprising six *indica*, three *aus*, two *rayada*, two *aromatic*, three *tropical japonica*, and four *temperate japonica* varieties. We used a stringent criterion to identify a total of 2886 high-confidence copy number variable regions (CNVRs), which span 10.28 Mb (or 2.69%) of the rice genome, overlapping 1321 genes. These genes were significantly enriched for specific biological functions involved in cell death, protein phosphorylation, and defense response. Transposable elements (TEs) and other repetitive sequences were identified in the majority of CNVRs. Chromosome 11 showed the greatest enrichment for CNVs. Of subspecies-specific CNVRs, 55.75% and 61.96% were observed in only one cultivar of ssp. *indica* and ssp. *japonica*, respectively. Some CNVs with high frequency differences among groups resided in genes underlying rice adaptation.

**Conclusions:**

Higher recombination rates and the presence of homologous gene clusters are probably predispositions for generation of the higher number of CNVs on chromosome 11 by non-allelic homologous recombination events. The subspecies-specific variants are enriched for rare alleles, which suggests that CNVs are relatively recent events that have arisen within breeding populations. A number of the CNVs identified in this study are candidates for generation of group-specific phenotypes.

## Background

In recent years, rice genomics has progressed substantially and generated considerable valuable resources, including availability of two independent genome sequences [1,2] and a composite physical map [3]. These resources provide a foundation for understanding the tremendous genetic diversity that exists in rice. Among organisms for which a high-quality genome sequence from at least one individual is available, such as human, mouse, and *Arabidopsis*, genome-wide surveys of single nucleotide polymorphisms (SNPs) have captured significant proportions of within-species variation [4-6]. Similarly, the rice research community has migrated to SNPs as the main measure of genetic variation in rice, with initiation of the OryzaSNP project (http://www.OryzaSNP.org), and subsequently ~160,000 nonredundant SNPs distributed across the entire genome of the OryzaSNPset have been discovered [7]. The abundant SNPs can be used to design more targeted SNP assays for immediate use in rice genetics and molecular breeding [8-10], particularly in Genome-wide association study (GWAS) [11-13].

However, genomics research has revealed other forms of genetic variations, such as copy number variation (CNV) in human [14,15], chimpanzee [16], dog [17,18], chicken [19], cattle [20], pig [21], rat [22], mouse [23], *Drosophila*[24], *Caenorhabditis elegans*[25], yeast [26], *Escherichia coli*[27], maize [28-30], and *Arabidopsis thaliana*[31,32]. CNV is defined as a segment of DNA ≥1 kb that is variable in copy number in comparison with a reference genome [33]. CNV covers more base-pairs [34-38] and has a higher per-locus mutation rate than SNPs [39]. Previous studies indicated that CNVs are in variable linkage disequilibrium (LD) with flanking SNPs [40-42].

High levels of CNV have been found throughout the rice genome [43,44], and recent study provided insight into the extent of genome-wide structural variations in the important representative restorer lines [45]. Herein we describe a more detailed survey undertaken to detect candidate CNVs in a panel of 20 Asian cultivated rice comprising six *indica*, three *aus*, two *rayada*, two *aromatic*, three *tropical japonica*, and four *temperate japonica* (Additional file 1: Table S1), and examine the genome-wide characteristics of CNVs in subspecies and groups. These resources allowed us to analyze genetic diversity as indicated by CNVs, to evaluate the biological roles of CNVs, and to identify candidate CNVs that are likely to occur independently in subspecies and contribute to the genetic differences among groups.

## Results

### Detection of CNVs in Asian cultivated rice

**Figure 1:**
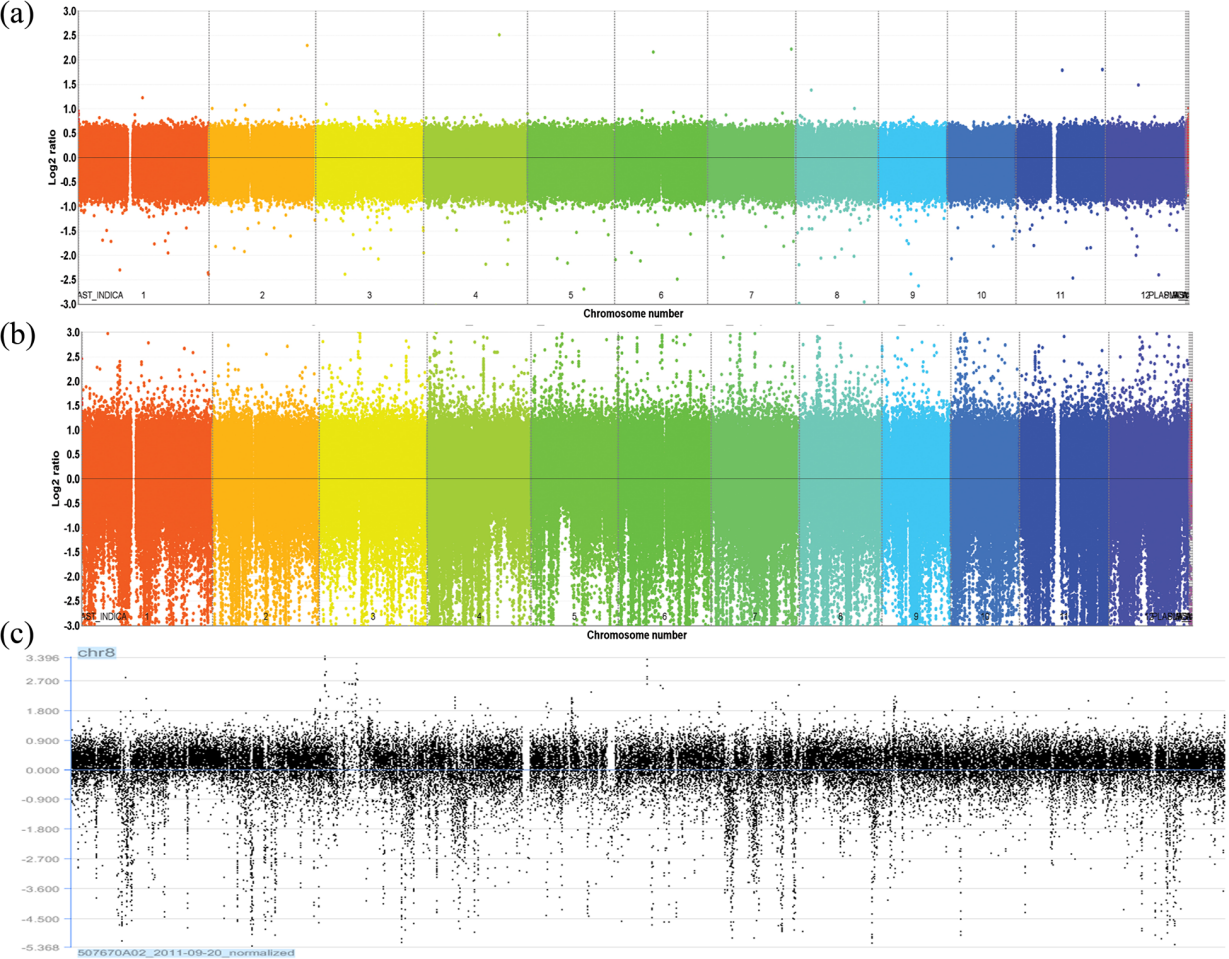
**Whole-genome DNA copy number quantification of the IR 64 by aCGH.** The linearized whole-genome copy number in shown in **(a)** Nipponbare (self–self hybridization), **(b)** IR 64, and **(c)** on chromosome 8 of IR 64.

**Figure 2:**
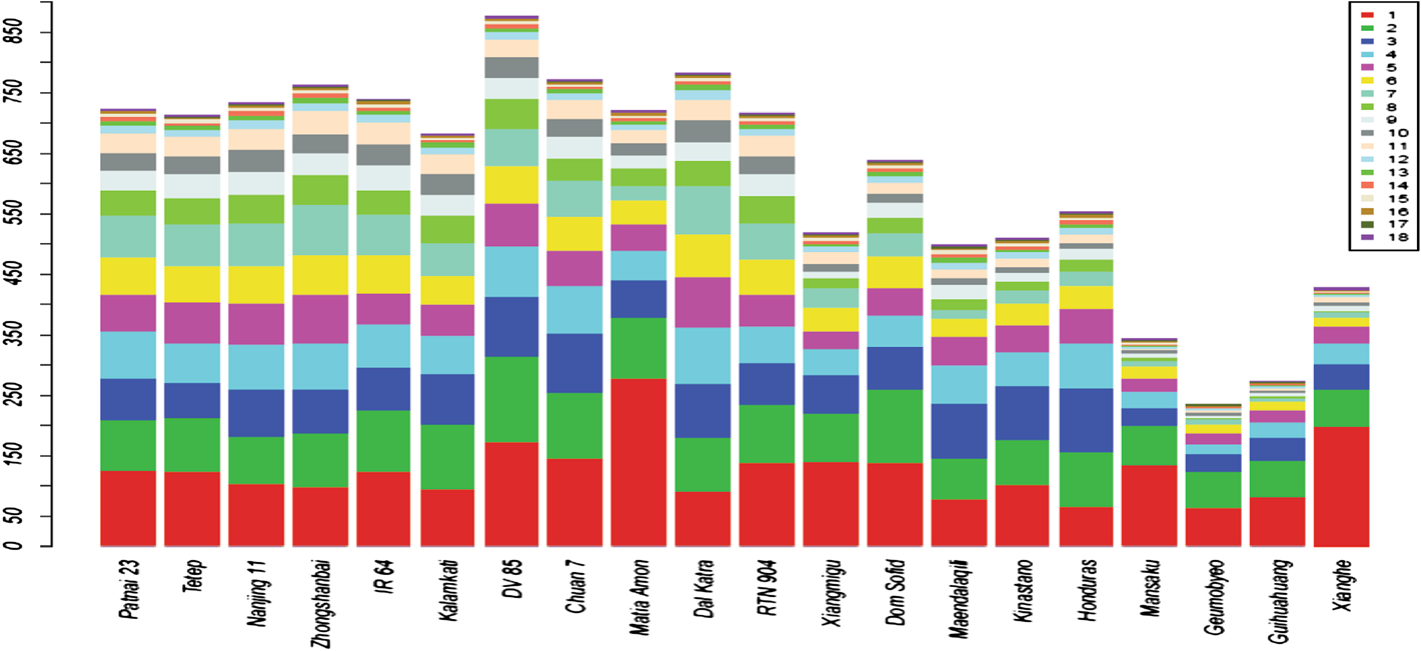
**Number of CNVs detected in each of 20 rice cultivars.** The numbers of cultivars carrying the CNVs are indicated by different colors.

We performed array-comparative genomic hybridization (aCGH) covering the entire rice reference genome sequence of ssp. *japonica* Nipponbare (IRGSP v4.0). DNA samples from Nipponbare, as the reference sample, were fixed in all hybridization experiments. Genomic DNA from 20 accessions of Asian cultivated rice was tested against the Nipponbare reference sample. We used an updated version of a previously described method to identify changes in log_2_ signal intensity corresponding to copy number gains and losses [43]. Under our conservative calling criteria, self-to-self hybridization showed no detectable false-positives. Figure 1 compares the linearized whole-genome karyogram of IR 64 to that of a self–self (Nipponbare), and shows the whole chromosome 8 in greater detail. Using a set of stringent criteria, a total of 12,224 CNVs in the 20 cultivars were identified, with an average of 611.2 per cultivar, ranging from 235 CNVs in Geumobyeo to 876 CNVs in DV 85 (Additional file 2: Table S2; Figure 2). A higher number of CNV events were detected in ssp. *indica* (746.8 per cultivar) than in ssp. *japonica* (500.3 per cultivar) (Table 1). However, among ssp. *japonica* cultivars, more CNV events were detected in *tropical japonica* (520.3 per cultivar) than in *temperate japonica* (320.0 per cultivar) (Additional file 2: Table S2). Analysis of variance (ANOVA) indicated that CNV length did not vary significantly among the cultivars (*F* = 0.551, *P* = 0.941), with 98.5% of the CNV lengths within 10 kb.

**Table 1:** Summary of CNVs identified in two rice subspecies

**Subspecies**	***N***^**a**^	**Total no. of CNVs**	**Average**^**b**^	**Range**^**c**^	**Gain**	**Loss**
ssp. *indica*	9	6721	746.8	683-876	543	6178
ssp. *japonica*	11	5503	500.3	235-783	310	5193

a Number of individual cultivars studied.

b Average number of CNVs per cultivar.

c Range in the number of CNVs identified per cultivar.

### Identification and distribution of copy number variable regions

By merging overlapping CNVs identified in all cultivars across the aCGH [15], 2886 high-confidence copy number variable regions (CNVRs) were identified, which covered 10.28 Mb of the rice genome (Additional file 3: Table S3). The CNVRs consisted of 2557 losses and 276 gains in copy number, and 53 with both events. Furthermore, 992 CNVRs (34.4%) were detected in only one cultivar (unique), whereas the remaining CNVRs (65.6%) were detected in two or more cultivars (Additional file 3: Table S3). However, more CNVRs were predicted to be present in ssp. *japonica* than in ssp. *indica* (Additional file 4: Table S4), which was inconsistent with the analysis of CNVs. The discrepancy may be attributable to the higher density of CNVs and fewer ssp. *indica* cultivars sampled.

**Figure 3:**
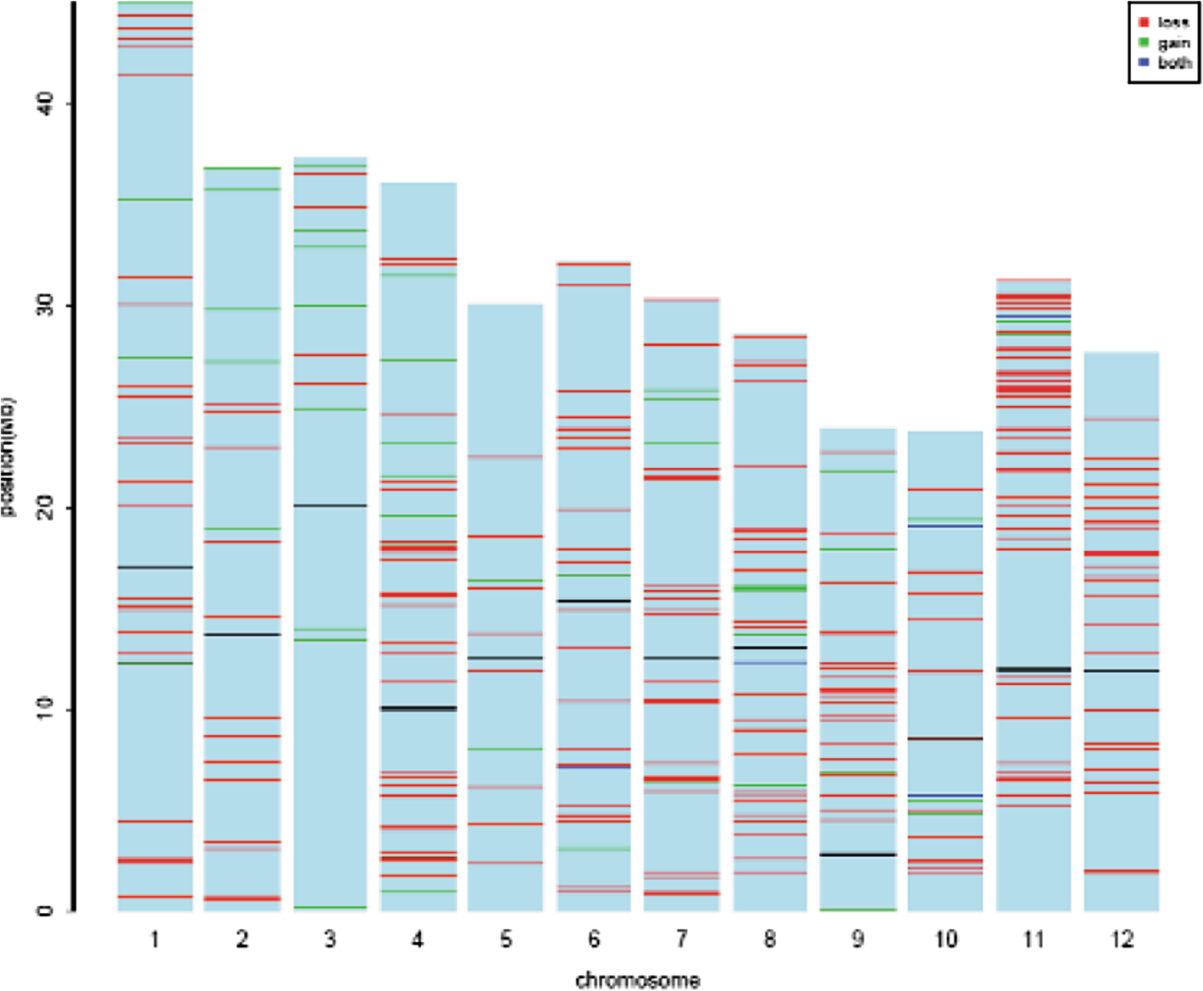
**Genome-wide distribution of CNVRs.** The ideograms depict the chromosomal location of copy number gains (green), losses (red), and gains or losses (blue) relative to Nipponbare in the 12 chromosomes of 20 rice cultivars. The approximate position of each centromere is indicated by a black line on each chromosome.

The CNVRs were distributed throughout all 12 rice chromosomes (Figure 3; Additional file 5: Table S5). The length of CNVRs differed significantly among different chromosomes, and the percentage of entire chromosomes susceptible to CNVRs ranged from 1.07% on chromosome 3 to 5.78% on chromosome 11. Although chromosome 11 spanned only 7.55% of the probes on the whole genome microarray, it included 17.54% of the CNVRs identified and thus showed the greatest enrichment for CNVs with ~2.15-fold the average variable content of the genome. There was little correlation between CNV occurrence and chromosome length (Additional file 5: Table S5), which is consistent with previous studies on the heterogeneous distribution of CNVs throughout the genome [15,46,47].

### Overlap of CNVs with segmental duplications (SDs) and other repeat contents

When we intersected the 2886 CNVRs with rice SD regions, 10.91% (315/2886) of the CNVRs directly overlapped with SDs. A reason is that only a unique sequence is spotted in the oligonucleotide array. This selectively omits or reduces the probe density in SDs. The regions identified as deletions relative to the reference sample were analyzed for repeat content (including transposable elements; TEs) for each chromosome (Table 2). The most common classes of TEs (long interspersed elements [LINEs], short interspersed elements [SINEs], long terminal repeat [LTR] retrotransposons, and DNA TEs) and other repetitive sequences, such as simple repeat sequences and other unclassified repeats, were identified in the majority of regions (2175/2557) (Additional file 6: Table S6). Of these regions, 639 contained LTR retrotransposons and 1080 contained no DNA TEs. Although differences in the number within each TE class at specific locations varied widely, LTR retrotransposons were the predominant class (45.1% of total TEs) followed by DNA TEs (almost 39.8% of total TEs). In addition, SINEs and LINEs were identified on all chromosomes and only in 6.1% (157) and 9.4% (240), respectively, of all deleted regions. The largest sequence length within the LINE signatures was 5540 bp. Chromosome 2 contained only five LINEs.

**Table 2:** Transposable elements (TE)/repeat contents of the deleted regions on each rice chromosome

**Chr**	**No. of deleted regions**	**Size (bp)**	**Total TEs/repeat contents (bp)**	**Repeats (%)**
1	208	676,382	11,9739	17.7
2	123	419,802	94,567	22.5
3	83	236,013	42,199	17.9
4	276	932,484	262,945	28.2
5	89	278,236	76,429	27.5
6	208	689,705	166,491	24.1
7	191	662,018	150,876	22.8
8	220	680,049	165,148	24.3
9	209	708,361	161,137	22.8
10	174	547,441	160,026	29.2
11	457	1,674,344	326,289	19.5
12	319	1,107,862	266,045	24.0

### Differentiation of subspecies by CNVs

**Figure 4:**
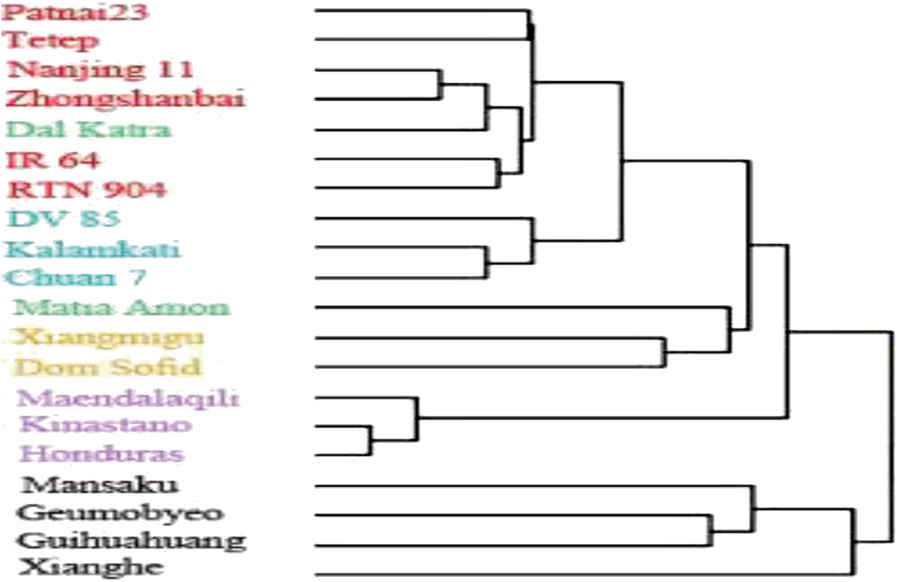
**Hierarchical clustering of the aCGH log**_**2 **_**ratio of all probes in 2886 CNVRs.** Cultivars are color-coded by group: *indica* in red, *aus* in blue, *rayada* in green, *aromatic* in orange, *tropical japonica* in purple, and *temperate japonica* in black.

Hierarchical clustering was performed to explore relationships among the 20 Asian cultivated rice based on the CNVs (Figure 4). Unsupervised clustering of the aCGH log_2_ ratio of all probes in the 2886 CNVRs supported known rice relationships and partially clustered samples consistent with previous studies [48], which indicated that those CNVs shared among samples may be indicators of a common evolutionary history and genetic relationship. However, the two rice subspecies, *indica* and *japonica*, did not form separate clusters. This result might be an artifact of the measurements being based on a common reference sample (Nipponbare), or the CNVs may be less phylogenetically reliable than neutral markers by reason of stronger selective constraints [49].

**Figure 5:**
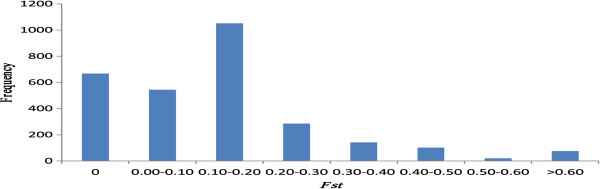
**Frequency distribution of *F***_**st **_**for all CNVRs.**

The approximate number of alleles was estimated using PowerMarker v3.25 software. Nei’s genetic diversity index (*H*_e_) value for ssp. *indica* and ssp. *japonica* at every polymorphic CNVR was 0.329 and 0.281, respectively. This was expected on the basis of previous findings that ssp. *indica* contains higher genetic diversity than ssp. *japonica*[50-53]. To better understand how CNV contributes to intra-subspecies diversity, we searched for CNVRs that exhibited high levels of heterozygosity and identified 14 CNVRs with higher diversity in one or both subspecies (*H*_e_ > 0.500). For example, one CNVR (chr10: 19103630–19117640) was identified to be polymorphic in both subspecies. We queried the gene content of this region; interestingly, it contained no known gene. The distribution of *F*_st_ across all CNVRs ranged from 0 to 1.000 (Figure 5), with an average *F*_st_ value of 0.138, which was slightly lower than estimates reported in previous studies in which global germplasm collections were used in combination with SNP or SSR data [11,12,48,54,55].

By further assessing the frequency of events separately observed in ssp. *indica* and ssp. *japonica*, 57.8% (1668/2886) of the variants were observed only in ssp. *indica* or ssp. *japonica*, but many of these were unique to one cultivar of ssp. *indica* or ssp. *japonica* (373/669 and 619/999, respectively). Only one CNVR (chr1: 35391036–35394841) was observed in all ssp. *indica* cultivars and no CNVR was observed in all ssp. *japonica* cultivars (Additional file 7: Table S7). This CNVR also overlapped in a previous aCGH analysis of Guangluai 4 and Nipponbare [43] and contained a subtilase gene Os01g0794800, a member of the superfamily of subtilisin-like serine proteases [56].

**Figure 6:**
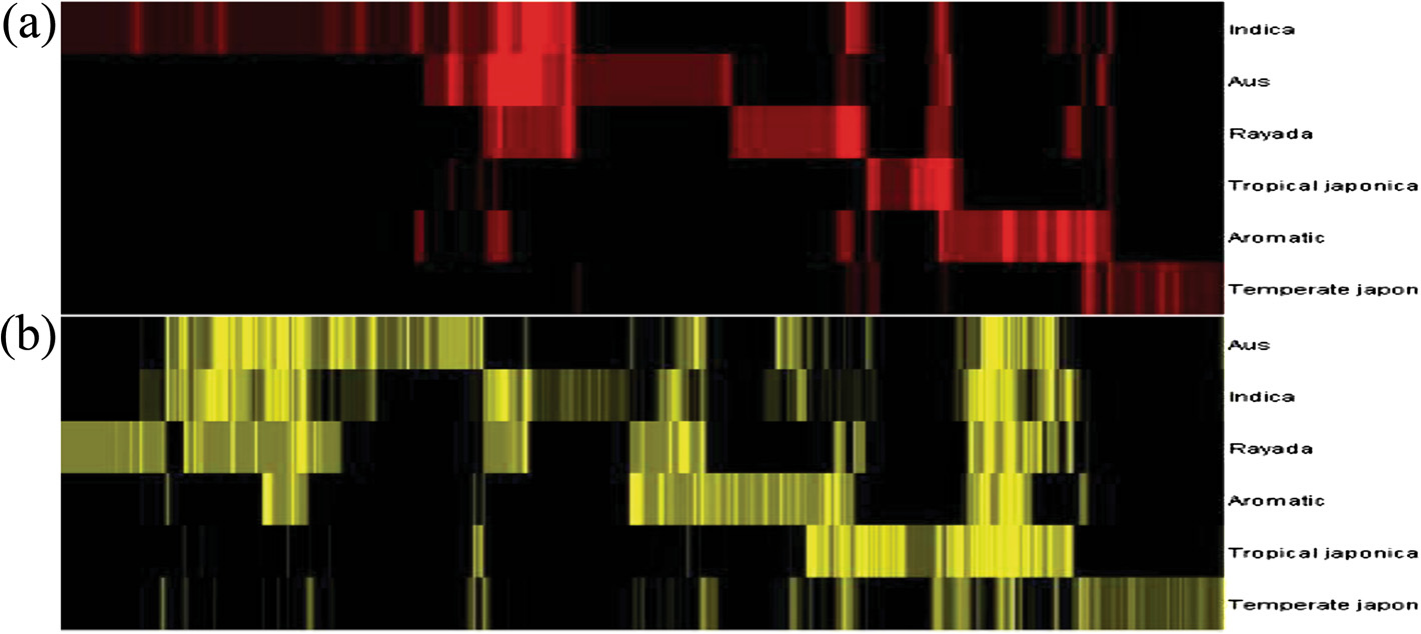
**Hierarchical clustering of both CNVRs and groups based on CNVR frequency within groups of rice cultivars.** The color indicates the type and frequency of each CNVR: red indicates amplification **(a)**, yellow indicates deletion **(b)**.

To visualize the distribution of both deletions and amplifications within the six groups of cultivars, event frequencies were analyzed by hierarchical clustering (Figure 6). The clustering identified variants that were restricted to certain groups and those that were present in multiple groups. Of 81 CNVRs with high frequency differences among groups (Additional file 8: Table S8), 54.32% corresponded to 51 annotated genes or gene families, of which some are important in rice adaptation, including a NB-ARC domain-containing protein. On the basis of differences in CNV frequency among the groups, we hypothesize that some CNVs arose independently within different groups and contribute to group differences, and therefore are associated with group formation and adaptation.

### Gene content of CNVs

**Figure 7:**
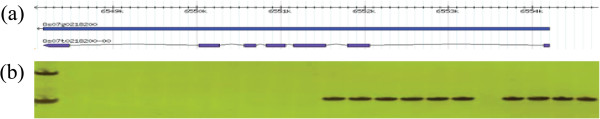
**Molecular confirmation of gene loss events detected by aCGH. (a)** The gene Os07g0218200 was encompassed entirely by a CNV (chr7: 6548063–6555290). **(b)** The two internal primers generated a 103 bp fragment; no product was detected by aCGH in the cultivars that contained the putative deletion. Individual lanes represent (left to right): DNA marker, Patnai 23, Tetep, Nanjing 11, Zhongshanbai, IR 64, Kalamkati, DV 85, Chuan 7, Matia Amon, Dal Katra, RTN 904, Xiangmigu, Dom Sofid, Maendalaqili, Kinastano, Honduras, Mansaku, Geumobyeo, Guihuahuang, Xianghe, and Nipponbare.

A total of 412 genes were identified as entirely affected by CNVs, which included 63 genes that were completely duplicated and 322 that were completely deleted. Five gene-loss events among the 20 Asian cultivated rice were chosen at random for PCR validation, namely Os01g0358700 (similar to the powdery mildew resistance protein PM3b), Os10g0507000 (NB-ARC domain-containing protein), Os11g0668650 (calmodulin-binding protein-like family protein), Os07g0218200 (similar to terpene synthase 7), and Os12g0247700 (similar to jasmonate-induced protein) (Additional file 9: Table S9; Figure 7). Given that the breakpoint definition of CNVRs can be equivocal, some of the gene sequences might partially overlap rather than be encompassed by CNVRs. Nevertheless, 1321 annotated genes wholly or partially spanned 41.6% (1200/2886) of the 2886 CNVRs (Additional file 10: Table S10), of which 46 (3.5%) were non-protein-coding transcripts. Overall we found that 70.0% (924/1321) of the genes identified were gene family members in the rice reference genome.

Gene ontology (GO) analysis of the 1321 overlapping annotated genes indicated that the functional classes of genes that were enriched within the 2886 CNVRs were cell death, protein phosphorylation, and defense response genes. We also identified orthologous genes of maize and *Arabidopsis* based on homolog clustering (Additional file 11: Table S11). The 523 rice-specific genes affected by CNV included 347 for which no additional homologs were found within rice and 176 that were in multigene families. The remaining 798 genes were conserved in maize and/or *Arabidopsis*.

## Discussion

### Characterization of CNVs in Asian cultivated rice

Several factors affected estimation of the number of CNVs detected between different rice cultivars against Nipponbare. First, the array designs target only the sequences present in the Nipponbare genome, and therefore we could not detect the sequences present in other rice cultivars but absent from Nipponbare. Second, a portion of the significant differences in hybridization signals may be due to the presence of multiple polymorphisms within the probe sequence between the reference and test sample genome. To reduce errors in CNV detection because of SNPs, we used a secondary manual method to examine all aberrant segments and to remove presumptive false-positives caused by single outlier probes and ensured all probes of each aberrant segment met a log_2_ ratio cut-off. As a consequence of this approach, our method for detection of CNVs was biased toward detection of small CNVs. Finally, self–self hybridization showed appreciable variability, but a set of conservative calling criteria for the final set of high-confidence CNVs showed no false-positives for self–self control hybridizations. Nonetheless, it is desirable to map the locations of the breakpoints with as high a degree of accuracy as possible.

We compared the 2886 CNVRs detected with 1676 candidate CNVs reported previously using re–sequencing results from 50 accessions of cultivated and wild rice [44]. The majority of the variants identified in these two studies do not overlap. In addition to the differences in detection technology, we suspect that the main reasons for this discordance are (1) differences in the accessions analyzed in the two studies, and (2) genome coverage biases—only unique sequences were spotted in our oligonucleotide array. This approach selectively omits or reduces probe density in CNV-enriched regions, such as SDs and diverse repetitive sequences [57]. In addition, it may also indicate that the CNVs detected using different technological and analytical platforms show large variation in CNV resolution, which may affect CNV sizes.

Previous studies have reported a strong association between CNVs and SDs in human [14,35,57-59], chimpanzee [16], mouse [23,60-62], and cattle [20]. In contrast, when we compared the 2886 CNVRs derived from aCGH data with rice SD regions, only 10.91% (315/2886) of the events overlapped with rice SDs. We suspect that the probe specificity in our arrays might severely interfere with CNV discovery. In the future, CGH arrays with unbiased genome coverage combined with improved CNV calling algorithms could remedy this imperfection.

A notable feature of the rice genome is that LTR retrotransposons were identified in 30.7% of the rice genome [63], which suggests that LTR retrotransposons may be an important source of CNVs in rice. Indeed, we performed a cursory analysis for TE/repeat contents in regions that were identified as being deletions relative to the reference sample. We identified 639 regions that contained LTR retrotransposons, which comprised over 45.1% of the total TEs identified in the deleted regions. Although these results are preliminary and require further analysis, they suggest that TEs and repeats may have played a role in shaping the genomic architecture of CNVs in rice.

Evidence for ancient whole-genome duplications in rice is reported [64,65]. The most recent segmental duplication event in rice occurred between chromosome 11 and 12 after differentiation of grasses [64]. This fact may explain why CNVR length differed significantly among different chromosomes, with larger CNVRs detected on chromosomes 11 and 12 in the present study. More surprisingly, previous work shows that chromosome 11 is enriched with disease resistance genes [66], which may be attributable to the higher number of CNVs on chromosome 11. Higher recombination rates and the presence of homologous gene clusters are probably predispositions for non-allelic homologous recombination (NAHR) events, which tend to generate CNVs [15,67].

### Differentiation of CNVs between ssp. *indica* and ssp. *japonica*

Rice is traditionally classified into two subspecies, ssp. *indica* and ssp. *japonica*, on the basis of morphological characteristics, ecological adaptation, crossing ability and geographic origin [68]. In addition, evidence suggests that ssp. *indica* and ssp. *japonica* are the products of separate domestication events from the ancestral species, *O. rufipogon*[69,70]. We investigated whether any CNVs are associated with ssp. *indica–*ssp. *japonica* differentiation. In our study, 57.8% (1668/2886) of variants were observed only in ssp. *indica* or ssp. *japonica*. However, many of these ssp. *indica-* and ssp. *japonica*-specific events were observed in only one cultivar (373/669 and 619/999, respectively). Therefore, the subspecies-specific variants are enriched for rare alleles and may represent relatively new events that have arisen within breeding populations. This is also consistent with the low proportion of long CNVs identified in our study. We hypothesize that these CNVs are associated with phenotypic diversity among rice cultivars, and further research is important to assess how these variants affect phenotype. We also searched for CNVs potentially associated with ssp. *indica–*ssp. *japonica* differentiation on the basis of high frequencies within ssp. *indica* and ssp. *japonica*. No variants were present in all ssp. *japonica* cultivars and not in any ssp. *indica* cultivar. Only one variant was observed in all ssp. *indica* cultivars and not in any ssp. *japonica* cultivar. Thus there was no evidence for strong effects of subspecies differentiation on structural variation. It should be noted that CNVs were documented based on comparison to a reference ssp. *japonica* genome, and therefore sequences present in the ssp. *indica* genome but not in the ssp. *japonica* genome were not detected. Thus, we speculate that subspecies differentiation-associated CNVs would be expected to be present in most ssp. *indica* cultivars, but in few or no ssp. *japonica* cultivars.

### Insights into Asian cultivated rice relationships based on high-density CNV data

Generally, the population structure of domesticated species is influenced by the evolutionary history of the predomesticated ancestors, as well as by the complexity of breeding practices [55]. Strong selective pressure during the process of rice domestication has led to the formation of population substructure [48,55,71]. In some studies, the two subspecies have been further divided into five major groups (*indica*, *aus*, *tropical japonica*, *temperate japonica*, and *aromatic*) on the basis of SNP and indel data [55,72]. In the present study, hierarchical clustering of CNVs indicated that the variation segregating with major rice groups was present in the samples of a rice group. However, smaller groups are adapted to specific ecosystems, which may be recognized as upland, deep water, or floating cultivars [71,73]. Evidence suggests that *rayada* is less amenable to *ex situ* conservation because of their adaptation to deep water. This may be one reason why the two *rayada* cultivars did not cluster together in the present study.

We conservatively queried CNVRs that have high-confidence frequency differences among groups. The allele frequencies that we observed for structural variants suggested that some variants have been removed entirely from certain groups. The specific regions may be related to diversification within specific rice groups. Further screening is necessary to confirm that these CNVs are truly group-specific. This finding also implies that those loss events detected exclusively in one group will limit the potential for genetic improvement through selection within that group only.

### Implications for biological roles for CNVs

It is generally assumed that individuals of the same species have very similar genome contents. Rice is autogamous and has relatively low nucleotide polymorphism rates compared to other crop species. However, the aCGH indicates that considerable genomic variation exists in Asian cultivated rice. Moreover, this variation may include substantial differences in gene content and gene structure, as was observed in the gene deletion analysis of the PCR data in the present study. In addition to 412 genes entirely encompassed by a CNV, some additional genes partially overlapped. This result could be explained by biological and technical factors. In addition to changes in gene dosage, the main mechanisms responsible for the potential effects of CNVs include reshaping of the gene structure and modification of the elements that regulate gene expression [74,75]. In addition, probe space and stringent criteria for CNV calling may limit coverage of the full length of genes. Notably, the array design is based on the older reference genome (IRGSP v4.0), so additional variant genes would not be identifiable if they reside in regions that were additions to the most recent version of the genome sequence. Collectively, these data suggest that the true number of genes may be substantially larger than the 1321 genes we identified in this study.

High levels of structural variation in plant genomes is related to important quantitative variation [30]. For example, fine mapping, complementation testing, and association analysis of a recently identified quantitative trait locus, *qSW5*, indicated that a deletion in *qSW5* resulted in a significant increase in sink size owing to an increase in cell number in the outer glume of the rice flower [76]. As was demonstrated in maize, CNVs are also important in plant disease responses by directly affecting causative genes [29]. Similarly, it is important to evaluate the effect of such structural variation on phenotypic plasticity in rice cultivars, groups and subspecies. Although there was no obvious phenotypic implication in the present study, a marked enrichment of genes for cell death, protein phosphorylation, and defense response may reflect the importance of CNVs in biological processes. We identified many disease resistance genes within the CNVRs. This is expected because, first, gene families with functions in regulatory processes and signal recognition, such as disease resistance, have higher nonsynonymous-to-synonymous substitution ratios [7,44]. This result, as for the over-represented set of disease resistance genes, might indicate a relaxation of constraints because of the redundancy expected from the variable number of gene copies [77,78]. Specifically, sequences encoding leucine-rich repeat and NB-ARC domains are common in plant disease resistance proteins, which are particularly diverse because of pathogen pressure [79-81]. Second, the loss of a single member of a gene family may result in a relatively minor loss of the total function of the gene family, because other family members may genetically cushion the impact. Thus, CNV is likely to contribute to quantitative variation rather than qualitative defects in the complex and highly duplicated plant genome [30]. In addition, the gene content in CNVs is speculated to have contributed to heterosis during domestication [28,30]. Indeed, high levels of variability in gene content among genotypes will result in hybrids that contain a higher number of genes than either parent. We observed in the present study that many of the rice cultivars were missing unequal numbers of genes relative to Nipponbare. It may be possible to identify a series of recombination events in order to combine all superior alleles for heterosis, which would be valuable in breeding programs.

## Conclusions

This study provides insights into the mutational mechanisms and functional effects of CNVs in the rice genome. Our results suggest that many CNVs are generated by NAHR events from higher recombination rates and the presence of homologous gene clusters, which is consistent with previous work that chromosome 11 is enriched with disease resistance genes. We also identify candidate CNVs for involvement in group-specific characteristics. This comprehensive catalogue of CNVs will be useful for future studies to uncover the genetic basis of complex traits in rice.

## Methods

### Oligonucleotide aCGH construction

A custom 3 × 720 k tiling-path aCGH for whole-genome analysis in *Oryza sativa* (IRGSP v4.0) was designed and constructed by NimbleGen Systems (http://www.nimblegen.com). Probes were synthesized using an isothermal format, varied in length from 50-mer to 75-mer, and spanned the rice genome with a median spacing of ~500 bp. The arrays were manufactured by maskless array synthesis technology, and the oligonucleotides were synthesized on the arrays by photolithography [82,83].

### Sample processing

We selected 20 rice cultivars to represent all of the major groups of *Oryza sativa* L. [48,71]. Their origins and features are summarized in Additional file 1: Table S1. The samples comprised two groups of ssp. *indica* (*indica* and *aus*) and four groups of ssp*. japonica* (*rayada*, *aromatic*, *tropical japonica* and *temperate japonica*). We also included the ssp. *japonica* Nipponbare, which was used to generate the rice reference genome sequence [3]. Genomic DNA was extracted and purified from fresh young leaves using the Plant Genomic DNA Kit (TianGen). DNA quality was assessed by measuring the concentration and purity with a NanoDrop ND-1000 spectrophotometer (NanoDrop Technologies). DNA integrity was assessed by electrophoresis in a 1.0% agarose gel. Standard genomic DNA labeling, hybridization, array scanning and intensity feature extraction were carried out as described previously [23,84] and performed by CapitalBio Corporation.

### Statistical analysis

Before normalization and segmentation analysis, spatial correction was applied, which corrected position-dependent non-uniformity of signals across the array. Locally weighted polynomial regression (LOESS) was used to adjust signal intensities based on *x*, *y* feature position [85]. Normalization was then performed using the *q*-spline method [86], compensating for inherent differences in signal between the two dyes. Segmentation analysis was performed with the segMNT algorithm in NimbleScan 2.5 software [87]. The segments were further filtered to remove all segments in which the average log_2_ ratio for all probes was > −2 or < 1 to produce a set of stringent segments. Aberrant segments were called deletions if the mean log_2_ ratio of probes in the segment was ≤ −2 and called amplifications with a mean log_2_ ratio ≥ 1. We used a secondary manual method to examine all aberrant segments and to remove presumptive false-positives caused by single outlier probes and ensure 100% probes of each aberrant segment meet the log_2_ ratio cut-off. In most cases these adjustments further decreased the count of the CNVs. We merged overlapping CNV coordinates across hybridizations to form unique CNVRs in accordance with similar criteria as described previously [15]. We queried the probes within the CNVRs against the most recent rice genome data release (IRGSP v5.0). It was possible to identify perfect matches (100% identity and 100% coverage) for 99.98% (715,698/715,851) of the probes for the 12 chromosomes. One hundred and fifty-three probes on the array no longer had perfect sequence matches in the genome, 99.92% of the probes (715,130/715,698) had only a single perfect match and were therefore deemed to be single copies, and 568 probes had more than two perfect matches. The probes were only retained if they had a unique optimal hit (100% sequence identity). The CNVRs that decreased the number of probes to less than five were discarded. Finally, the remaining CNVRs were retained if they did not overlap a large gap in IRGSP v5.0.

We inferred approximate allele frequencies by simplifying CNV phenotypes into three categories: normal, loss, and gain. The frequency of each category was estimated by PowerMarker 3.25 software [88]. The estimated allele frequencies were used to calculate heterozygosity (*H*_e_) [89] for each subspecies and each polymorphic CNVR. Similarly, for each CNVR we calculated *F*_st_ as *F*_st_ = 1− *H*_s_/*H*_t_, where *H*_s_ and *H*_t_ denote average heterozygosity within subspecies and total heterozygosity, respectively.

All probe signals of each CNVR were subjected to unbiased clustering for the 20 rice cultivars using average linkage and correlation (uncentered) as the metric. The frequency of CNVs within each group was analyzed using hierarchical clustering. The single case where we identified loss and gain within the same region could have been treated as a multistate locus, but instead we chose to exclude complex events from this analysis. ANOVA was performed with SPSS 16.0.

### Functional characterization of genes affected by CNVs

The gene content of CNVRs was examined using the IRGSP v5.0 gene models (http://rgp.dna.affrc.go.jp/IRGSP/Build5/build5.html). GO annotation from RAP-DB (http://rapdb.dna.affrc.go.jp) of genes that were affected by CNVs was assessed using the Gene Ontology Enrichment Analysis Software Toolkit (GOEAST) [90]. *P*-values for enrichment were calculated using a hypergeometric test method with false discovery rate correction [91]. Of the 42,081 IRGSP v5.0 gene models used, 15,689 (37.28%) had GO terms from RAP-DB (http://rapdb.dna.affrc.go.jp). Of the 1321 gene models in CNVRs, 561 (42.47%) had GO annotations that were used in our GO enrichment analysis.

Genes belonging to gene families across the genome were separated from a single-copy gene set according to http://green.dna.affrc.go.jp/PGF-DB/. The rice-specific genes and gene families were identified based on homolog clustering with annotated genes of maize and *Arabidopsis* using the method previously described by McGinnis and Madden [92].

### Analysis of TEs and repeats in the deleted regions relative to Nipponbare

The regions identified as deletions relative to the reference sample were analyzed for TEs and repeats. Repeats were detected using R_EPEAT_M_ASKER_ (http://www.repeatmasker.org). Repeat content was expressed as the percentage of nucleotides masked versus the total.

### PCR validation

Gene loss events were assessed by PCR. Amplifications were performed on a 2720 Thermal Cycler (Applied Biosystems) under standard conditions for 30 cycles. The PCR products were electrophoresed on 6% non-denaturing polyacrylamide gel.

### Access to data

The full data set from the oligonucleotide aCGH experiments has been submitted to the NCBI Gene Expression Omnibus (http://www.ncbi.nlm.nih.gov/geo) [93] under the accession ID GSE42769.

## Abbreviations

CNV: Copy number variant; CNVR: Copy number variable region; TE: Transposable element; SNP: Single-nucleotide polymorphism; LD: Linkage disequilibrium; GWAS: Genome-wide association studies; aCGH: array comparative genomic hybridization; SD: Segmental duplication; LINE: Long interspersed nuclear element; SINE: Short interspersed nuclear element; LTR: Long terminal repeat; GO: Gene ontology; NAHR: Non-allelic homologous recombination.

## Competing interests

The authors declare that they have no competing interests.

## Authors’ contributions

PY and XHW conceived and designed the experiments. YF, XPY, HYY and YPW performed the experiments. PY, CHW, and QX contributed to the interpretation of the data. PY, XHW, and SXT drafted the manuscript. All authors read and approved the final manuscript.

## Supplementary Material

Additional file 1: Table S1 Selection of rice samples. Click here for file

Additional file 2: Table S2 Sample level CNV calls from aCGH. Click here for file

Additional file 3: Table S3 IRGSP v5.0 CNVRs and their frequencies. Click here for file

Additional file 4: Table S4 CNVRs in ssp. *indica* and ssp. *japonica.* Click here for file

Additional file 5: Table S5 CNVR coverage in all chromosomes. Click here for file

Additional file 6: Table S6 Locations and classifications of repeats found in the deletions relative to the reference sample. Click here for file

Additional file 7: Table S7 CNV frequency difference between subspecies. Click here for file

Additional file 8: Table S8 High confidence CNVR frequency differences among groups. Click here for file

Additional file 9: Table S9 Primers used for PCR validation of gene loss events. Click here for file

Additional file 10: Table S10 Gene content of 2886 CNVRs. Click here for file

Additional file 11: Table S11 Conservation analysis of 1321 genes in *Arabidopsis* and maize. Click here for file
